# Psychometric properties of the Persian version of the Physical Activity Scale for the Elderly (PASE)

**DOI:** 10.1186/s12877-021-02337-0

**Published:** 2021-06-23

**Authors:** Omid Hatami, Mahdi Aghabagheri, Somayeh Kahdouei, Khadijeh Nasiriani

**Affiliations:** 1 Department of Nursing, Research Center for Nursing and Midwifery Care, Shahid Sadoughi University of Medical Sciences and Health Services, Yazd, Iran; 2 Medicine School, Shahid Sadoughi University of Medical Sciences, Yazd, Iran; 3 Faculty of Psychology, Imam Javad University, Yazd, Iran; 4 Department of Nursing, Mother and Newborn Health Research Center, Research Center for Nursing and Midwifery Care, Shahid Sadoughi University of Medical Sciences, Yazd, Iran

**Keywords:** Ageing, Physical Activity, Psychometrics, PASE, Persian, Farsi

## Abstract

**Background:**

Old age is associated with reduced physical ability. It is necessary to measure and evaluate the physical activity of older people. Implementing appropriate requires a valid and reliable tool. Physical Activity Scale for the Elderly (PASE) is the frequently used self-reported physical activity assessment for older adults. Therefore, this study aimed to determine the translation validity and reliability of the Persian version of the Physical Activity Scale for the Elderly.

**Methods:**

This study is a methodological, descriptive applied research was conducted on 300 older people. The translation process of the English version of PASE into Persian was carried out according to the process of translation and adaptation of scale recommended by the World Health Organization. The reliability of the scale was examined by calculating the Cronbach’s alpha, Pearson, and intraclass correlation coefficient (ICC). The reliability and validity of the scale were evaluated by following the “Consensus-Based Standards for the Selection of Health Status Measurement Instruments” (COSMIN) checklist. To assess the face and content validity, impact score (IS), the content validity ratio (CVR), and the content validity index (CVI) were determined. A confirmatory factor analysis was also performed.

**Results:**

The experts approved the quality of the Persian version of PASE. The reliability was calculated with a Cronbach’s alpha of 0.94, an ICC of 0.99, and a test-retest correlation coefficient of 0.94. The qualitative and quantitative face validity of all questions by expert judgment and IS of greater than 1.5 was considered. Also, CVR and CVI scores of all questions were higher than 0.6 and 0.79, respectively. Confirmatory factor analysis revealed a good fit for the original three-factor structure.

**Conclusions:**

The Persian or Farsi version of PASE was shown to have acceptable validity and reliability. This tool is suitable for measuring the physical activity level in the Persian elderly language special in clinical environments and therapeutic interventions.

## Background

Ageing is one of the most important anthropological phenomena of the century [1]. The life expectancy of older people is rising around the world [2]. In Iran, in the next decade, an increase in the population of the elderly will be significant, that the population of people over 60 will reach 8.5 million [3]. Ageing is accompanied by a reduction in physical abilities in all countries [4]. Also, the rate of motor disability in the Iranian elderly is significant [5].

Today, the issue of improving older people’s health and their physical activity in the older ages is a public health problem and a serious issue [6]. On the other hand, the factors affecting longevity, quality of life, life expectancy, life satisfaction, the feeling of being good, lifestyle, and regular physical activity are known today [7]. Although barriers to exercise were multiple and complex including the gender barriers, role of habit in exercising, cultural infrastructure and fear of catching a disease or recurrence of an illness [8]. Also, regular physical activity in old age can prevent osteoporosis and its consequences such as femoral and pelvic bone fractures, obesity, depression and colon cancer, and, in general, the biological changes associated with ageing [9, 10]. Having an active life makes older people healthy and independent [11]. Regular physical activity, such as walking, reduces dependence on other family members and caregivers, accelerates socialization and, improves mental health [12–16], while immobility is one of the top 10 causes of death in the world [17]. An inactive life is among the main causes of chronic diseases such as cardiovascular disease, diabetes mellitus, etc. [18].

There are several tools for measuring daily activity in adult populations, which are also used for the elderly. Some of them are Functional Independence Measure (FMI), Barthel index and Katz Activities of Daily Living (ADL) [19, 20]. Although these tools, have been good metrics proprieties and are widely used to assess functional decline [19] some of them, such as the Katz Index used to assess the level of independence in older adults [20]. Specifically, most activity questionnaires do assess moderate-to-vigorous physical activity and not involved in less strenuous activities such as light housework [21, 22]. Also, little is known about the patterns of physical activity among older people [23, 24]. This is due, in part, to a lack of reliable and valid measures of physical activity among the aged [21]. One of the tools for measuring the level of physical activity in the elderly, currently used globally, is the Physical Activity Scale for the Elderly (PASE). PASE was first designed in 1993 by Washburn et al. [25]. The PASE is a self-report/interview-based measure designed to capture and assess occupational, household, and leisure activities typically performed by older adults; that is, those who are 65 year. of age and older [21]. The results indicated that PASE can be considered as an appropriate tool for measuring the physical activity of the elderly [25]. The advantages of The PASE compared to the others are the short practice period, the easy scoring process, and self-report/interview-based measures that applicability via letters or phone [21]. Separately, it consists of three subheadings of leisure time, household, and work-related activities [25]. which makes it easier to compare subheadings with others and to evaluate the physical activities of individuals among themselves in more detail [26, 27]. To investigate the broad characteristics of physical activity of the Persian population, the translation and validation of scale are important. Therefore, the aims of the study were to translate the PASE scale into Persian, to culturally adapt the instrument, and to evaluate its validity and reliability.

## Methods

This study is a methodological, descriptive, applied research that evaluates psychometric properties. This psychometric study was performed in three phases of the translation and cultural adaptation, reliability, and validity of the scale. The translation and adaptation of instrument followed the process of translation of the world health organization [28]. The reliability and validity of the culturally adjusted scale were evaluated by following the “Consensus-Based Standards for the Selection of Health Status Measurement Instruments” (COSMIN) checklist [29].

### Instrument

The PASE originally developed in the United Kingdom in 1993. The PASE measures the level of self-reported physical activity in individuals aged 65 years or older for the purpose of assessing the components of physical activities involving leisure time, work-related activities, and the household during the previous 7-day period [21]. The PASE evaluates the frequency, duration, and intensity of physical activities related to walking; light, moderate, and strenuous sports and entertainment activities; muscle strengthening and endurance exercises; work-related activities including walking and standing up; lawn and garden care; care for another individual; house repairs; and heavy and light household activities. The questions are scored differently. The total PASE score is computed by multiplying either the time spent in each activity (hours per week) or participation (i.e., yes/no) in an activity, by empirically derived item weights and then summing overall activities. The overall PASE score ranges from 0 to 400 or more and high scores show better physical activity levels [26, 27]. The English version of PASE is available at the following web address: https://www.physio-pedia.com/Physical_Activity_Scale_for_the_Elderly_(PASE). The PASE administration and Scoring Instruction Manual is available at the following web address: https://meetinstrumentenzorg.nl/wp-content/uploads/instrumenten/PASE-handl.pdf.

### Participants

The participants of the study had to be aged 65 years and older, based on previous validations of the PASE [29]. Three hundred elder people were selected from the retirement community or the City Pensioners Association by convenience sampling in Yazd province, Iran. The inclusion criteria for older people were able to perform daily activities independently and were mobile and willingness to cooperate. Exclusion criterion was amputation in the upper and lower limbs, having some diseases or progressive illness (such as rheumatoid arthritis, cancer, serious osteoporosis, stroke, and severe cardiovascular disease), neurological or general diseases, and mental illness with medical treatment that could have negatively influenced the daily life activity. Inclusion and exclusion criteria were assessed by reviewing the file and asking questions of the participants.

### The translation and cultural adaptation phase

The first phase was related to the translation and cultural adaptation of scale. In this phase, the scale was translated based on the process of translation and adaptation of scale recommended by the World Health Organization. The purpose of this process is to achieve different language versions of the English tool that are conceptually equivalent in each of the target nations/cultures. The tool, while simple and practically acceptable perform in the same way, should be equally natural and acceptable. The focus in this method was on cross-cultural and conceptual nature, rather than on linguistic meanings or literal equivalence. A well-established process to achieve this goal is to use forward-translations and back-translations. The implementation of this method includes the four steps included forward translation, expert panel back-translation, pre-testing and cognitive interviewing, and final version. In this study, first, the scale was translated into Persian by two expert translators (health professionals, familiar with the terminology of the area and English-speaking culture). Then was established an expert panel with two English experts, two geriatric experts, and two physical activity experts. They were asked to study the scale carefully and compare it to the original version in terms of it being equivalent in meaning. Thus it was produced a complete translated version of the scale. After this stage, the translated scale was translated into English by two experts in English without access to the original scale. The pre-test and cognitive interview respondents were ten older people. Then the final edition scale for the rest of the study was confirmed.

### The reliability phase

The second step was related to the reliability of the scale. Reliability refers to the consistency or repeatability of the measure. A Cronbach’s alpha higher than 0.7 was considered reliable [30].

The internal reliability of the scale was determined through Cronbach’s alpha coefficient. The test-retest reliability was calculated Intraclass Correlation Coefficient (ICC) and Pearson’s correlation coefficient for 20 elders with a two-week interval. The agreement between a measurement applied to a sample of individuals and the same measurement repeated later .The usual test–retest interval is between 10 and 14 days [31, 32].The elders selected from the retirement community by simple random sampling. A list of seniors over the age of 65 was obtained from the manager.then, using a computer, 20 subjects were randomly selected from among the members of retirement community. The phone number and postal address of the selected person was taken from the manager. The scale was completed according to the opinion of the elderly at home or retirement community center. An ICC between 0.6 and 0.8, and higher was regarded as good and excellent, respectively [33, 34].

### The validity phase

The third phase related to the validation of the scale that included face (qualitative and quantitative) and content validity. Purposive sampling was also used in relation to the determination of face and content validity as well as the selection of experts in the field of geriatric and the selection of older people (10 people).

In this study to determining qualitative face validity, the opinions of a 6-person specialist panel including two professional health, two sports, and two geriatric experts, were considered. The level of difficulty, ambiguity, and vague expressions, or any difficulty with comprehension and understanding of the concepts was checked. The corrective comments of this stage were examined and reviewed in a panel consisting of members of the research team and other invited experts.

The quantitative face validity was examined by calculation of Impact Score (IS) for each item within the questionnaire. A Likert scale with 5 options and scores of 1–5 was measured and evaluated. The range of options contains: Extremely important (score 5), very important (score 4), moderately important (score 3), slightly important (score 2), and not at all important (score 1). IS for each item was calculated as multiplying the importance of an item with its frequency. The impact scores of greater than 1.5 were considered suitable and item chosen for further analysis. The IS of greater than 1.5 were considered suitable and item chosen for further analysis [35, 36]. In this study, the scale was distributed among 10 older people and the IS was calculated for each question.

In order to assess the qualitative content validity of the translated scale, five experts (geriatric, physical activity, and community health nurse) were asked to study the tool carefully and provide written corrective comments on grammar, vocabulary, and the use of proper words, the importance of questions, proper order of questions, and the time to complete the questionnaire. The corrective comments of this stage were examined and reviewed in a panel consisting of members of the research team and other invited experts.

Content validity is defined as the degree to which items of a tool are relevant to and representative of the targeted concept for a specific evaluation purpose [37]. In the current study, quantitative content validity was assessed two different methods were used to check the content validity of the scale: Content Validity Ratio (CVR) and Content Validity Index (CVI).

To determine CVR, 10 experts were asked to check each of the items of the scale each item was scored according to three points (1 = not necessary, 2 = useful, but not essential, and 3 = essential). The formula of CVR is CVR=(N_e_ - N/2)/(N/2), in which the N_e_ is the number of panelists indicating “essential” and N is the total number of panelists [38]. Based on Lawshe Table, if‏ the number of panelists was ten panelists, the CVR score is higher than 0.6 .the item on the scale with an acceptable level of significance‏ will‏ be‏ approved [39].

CVI is the most commonly used method to calculate content validity quantitatively [40]. To this end, panel experts are asked to rate scale items in terms of relevance or specificity. The relevance or specificity of each item was categorized with the following options of 1 (not relevant), 2 (somewhat relevant), 3 (quite relevant), and 4 (very relevant). CVI is computed as the number of experts giving a rating of “very relevant” for each item divided by the total number of experts. The CVI was calculated based on the Waltz and Bausell [41].Values range from 0 to 1 where CVI > 0.79, the item is relevant and accepted, between 0.70 and 0.79, the item needs revisions, and if the value is below 0.70 the item is eliminated [40, 42].

The Confirmatory Factor Analysis (CFA) was carried out to determine the factor structures of the Persian version of PASE. The models were compared with each other according to the obtained dispersion indices including the ratio of Chi Square to its degrees of freedom (χ2/df), Comparative Fit Index (CFI), Goodness Of Fit Index (GFI), Adjusted Goodness Of Fit Index (AGFI), Root Mean Square Residual (RMR), and Root Mean Square Error of Approximation (RMSEA) [43].

### Statistical analysis

The statistical methods used in this study were descriptive and inferential statistics performed using Microsoft Excel 2013 for computing IS, CVR and CVI. The SPSS version 16 used for calculation Kolmogorov-Smirnov, Cronbach’s alpha, ICC, and Pearson’s test. The AMOS software version 20 was used to perform the confirmatory factor analysis of the PASE.

### Ethical consideration

 This study was approved by the ethical code IRSSU.REC.1395.154 from Shahid Sadoughi University of Medical Sciences in Yazd. Also, in all phases, consent was received from the participants including faculty and expert members and the older people.

## Results

Based on the results, the majority of participants were male, married, and dimple and higher. The characteristics are reported in Table 1. Also, a one-sample Kolmogorov-Smirnov test of normality showed that the data was normally distributed (high-grade, *p* = 0.2; low-grade, *p* = 0.2).

**Table 1:** Frequency distribution of demographic characteristics of participants

Demographic characteristics		Number	Percent
Sex	Female	98	32.7
	Male	202	67.3
age	65–70	153	51
	71–75	73	24
	≥ 76	74	25
Marital status	Single	75	25
	Married	225	75
Education	Illiterate	67	22.3
	Under diploma	72	24
	diploma and higher	161	53.7
Type of living	Living with others	257	85.7
	Living alone	43	14.3
Employment status	Full-time	9	3
	Part-time	87	29
	Unemployed	204	68
total		300	100

The Cronbach’s alpha and ICC values were used for estimating the internal consistency of the scale. The Cronbach’s alpha was 0.94 for the whole scale. ICC value was obtained with a confidence interval of 0.95 was 0.97, which was all statistically significant (*P* = 0.0001). Also, in the test-retest, the Pearson’s correlation coefficient was 0.94 for the total scale (Table 2).

**Table 2:** Cronbach’s alpha, ICC, Pearson’s test for reliability of PASE

Dimensions PESE	Mean ± Standard Deviation	Cronbach’s alpha	ICC	*P*.VALUE	Pearson’s test
Leisure time activities	30.77 ± 13.90	0.86	0.95	0.0001	*r* = 0.92
House hold activities	73.52 ± 41.45	0.97	0.89	0.0001	*r* = 0.94
Work-related activities	28.37 ± 15.05	0.99	0.97	0.0001	*r* = 0.96
Total score	132.67 ± 53.28	0.94	0.93	0.0001	*r* = 0.94

At the beginning of the study, the questionnaire was translated in a standard manner in several stages. After verifying the accuracy of the translation, the qualitative face validity of the Persian version of PASE was confirmed by experts’ judgment. Then, quantitative face validity was confirmed through the IS of each question answered by the older people. The questions with IS of equal to or greater than 1.5 were included in the questionnaire and the 7th question was revised based on the results based on the climatic and ethnic conditions of Iran, relatively intense activities were added to this question. So, all questions of scale were accepted in this phase. In assessing the qualitative content validity of the Persian version of PASE was confirmed by applying experts’ opinions as follows: “voluntary” was changed to “charity”, “how many hours per week did you work for pay and or as a volunteer” was changed to “last week, did you work for a charity or for pay”. Content validity ratio (CVR) and content validity index (CVI) was used for quantitative evaluation of content validity. Result CVR showed all questions, gained an acceptable score, and was thus confirmed. Based on Waltz and Bausell’s CVI with a score of 0.79 and higher was acceptable. Therefore, all of the questions have acceptable validity (Table 3).

**Table 3:** IS, CVR, and CVI of PASE

Question	IS	CVR	CVI
Recreational activities			
1. Over the past 7 days, how often did you participate in sitting activities such as reading, watching TV, or doing handcrafts?	3.96	0.8	0.86
(b) On average, how many hours did you engage in these sitting activities?	4.05	1	0.93
2. Over the past 7 days, how often did you take a walk outside your home or yard for any reason? For example, for fun or exercise, walking to work, etc.	4.7	1	0.86
(a) On average, how many hours per day did you spend walking?	4.6	1	0.86
(b) How much distance did you cover over the past 7 days? (1.5 km or 1500 m)	2.87	0.7	0.8
3. How many floors did you climb over the past 7 days? (each floor = 10 steps)	4.6	0.7	0.86
4. Over the past 7 days, how often did you engage in moderate sport and recreational activities such as walking, mild running, light swimming or other similar activities?	3.28	1	1
(b) On average, how many hours a day did you engage in these moderate sport or recreational activities?	3.36	0.8	0.8
5. Over the past 7 days, how often did you engage in moderate sport and recreational activities such as ping pong, recreational volleyball or other similar activities?	3.69	0.7	1
(b) On average, how many hours a day did you engage in these moderate sport or recreational activities?	2.59	0.7	0.83
6. Over the past 7 days, how often did you engage in strenuous sport and recreational activities such as jogging, swimming, cycling, singles tennis, aerobic exercise, mountaineering or other similar activities?	2.1	0.7	0.86
(b) On average, how many hours a day did you engage in these strenuous sports or recreational activities?	1.98	0.8	0.46
7. Over the past 7 days, how often did you do any exercises specifically to increase muscle strength and endurance, such as lifting weights or push up, etc.?	1.8	0.8	0.86
(b) On average, how many hours a day did you engage in these strenuous sport activities or relatively intense activities?	1.5	0.68	0.79
Household activity			
8. During the past 7 days, have you done any light housework, such as dusting or washing dishes?	4.8	0.7	1
9. During the past 7 days, have you done any heavy housework or chores, such as vacuuming, scrubbing floors or washing windows?	3.96	0.7	0.8
10. During the past 7 days, did you engage in any of the following activities?			
a. Home repairs like painting, wallpapering, electrical work, etc.	2.8	0.8	0.86
b. Lawn work or yard care including leaf removal, car repair, shopping, etc.	4.7	0.8	0.8
c. Outdoor gardening	2.22	0.8	0.93
d. Caring for another person, such as children, dependent spouse, or another adult	5	0.8	0.8
Work-related activity: 11. During the past 7 days, did you work for pay or as a volunteer?	2.87	1	0.8
a. How many hours per week did you work for pay and or as a volunteer?	3.28	1	0.8
b. Which of the following categories best describes the amount of physical activity required on your job and or volunteer work?			
1. Mainly sitting with some slight arm movement (Examples: office worker, watchmaker, seated assembly line worker, bus driver, etc.) 2. Sitting or standing with some walking (Examples: cashier, general office worker, light tool and machinery worker) 3. Walking with some handling of materials generally weighing less than 25 kg (Examples: mailman, waiter/waitress, construction worker, heavy tool and machinery worker) 4. Walking and heavy manual work often requiring handling of materials weighting over 25 kg (Examples: lumberjack, stone mason, farm or general laborer)	3.36	1	0.86

**Fig. 1:**
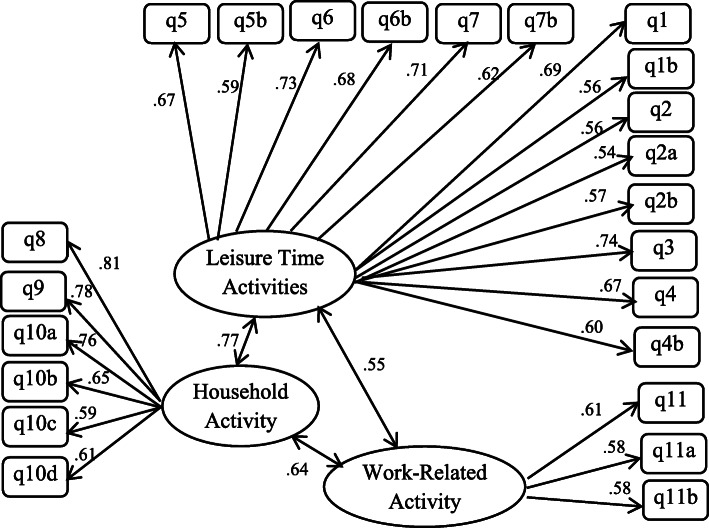
Confirmatory factor analysis model

Construct validity was examined by conducting a confirmatory factor analysis. The values ​​of fitness indices of the scale show that except for the normed fit index (NFI), the other indices are acceptable. This indicates that the model of measurement is fit (Table 4). Also, All path coefficients were significant (*p* < 0.01) (Fig. 1).

**Table 4:** Fitness indices for the primary PASE model

Index grouping	Index name	Abbreviation	Primary model	Acceptable fit
Absolute fit indices	Level covered by chi-square	χ2	137.01	-
	Degree of freedom	DF	59	-
	Significance level	P	0	> 0.05
	Goodness of fit index	GFI	0.937	GFI > 0.90
	Adjusted goodness of fit index	AGFI	0.902	AGFI > 0.90
Comparative fit indices	Non-normed fit index	NNFI	-	NNFI > 0.90
	Normed fit index	NFI	0.86	NFI > 0.90
	Comparative fit index	CFI	0.917	CFI > 0.90
	Incremental fit index	IFI	0.919	IFI > 0.90
Parsimonious fit index	Parsimonious normed fit index	PNFI	0.655	Greater than 0.5
	Root mean square error of approximation	RMSEA	0.066	RMSEA < 0.10
	Normed chi square	CMIN/DF	2.322	between 1 and 3
	Root mean square residual	RMR	0.08	< 0.05

## Discussion

The purpose of this study was to assess the validity and reliability of the Persian version of PASE. The present study was conducted on 300 elderly people living in Yazd, Iran who had no sever cognitive and physical disability. The results showed that the translation had a desirable quality. Specialized language and ageing experts and elder people approved the quality of the translated version.

The psychometric properties of PASE included reliability, face, and content, construct validity. The reliability of confirmed by Cronbach’s alpha, ICC, and Pearson’s test. In terms of qualitative face validity, the scale was approved with regard to the appearance of questions. IS was showed a quantitative acceptable score of face validity. The qualitative content validity has confirmed by an expert panel. The CVI and CVR score showed acceptable content validity. Confirmatory Factor analyses revealed three factors: leisure time activities, house hold activities, and work-related activities.

In this study, translated the PASE Persian and adapted it to Persian culture. So, PASE is a simple, valid, and reliable scoring tool for physical activity in the Persian elderly language and useful sale for assessing the physical activity of the elderly in research and clinical environments. Other studies showed PASE scale is valid in English and has been studied in many countries. The Chinese, Turkish, Japanese, Italian, etc. versions have also been adopted [25, 26, 29, 44–46]. However, the results are inconsistent for the elderly with special diseases, PASE is considered a valid tool for self-reporting physical activity in patients with lung cancer [45]. Svege showed the reliability of the test-test and construct validity showed that PASE did not have the ability to examine such physical activities in patients with osteoarthritis [47]. Therefore, in future studies, it is necessary to examine this tool in the physical activity of the elderly with the disease.

One of the limitations was the samples of this study were retired elderly members of the Retirement Association; the samples cannot reflect the Persian general population, which should be considered in order to generalize the findings to the entire elderly community. Another limitation of the study was that Iran is a vast country and its provinces have different climatic conditions that affect the physical activity of the elderly. In this study, psychometric measurements were performed in areas with hot and dry climates. On the other hand, The PASE score combines information on leisure, household and occupational activity that can be influenced by climatic and environmental factors, cultural and social conditions and place of residence. So, The Persian version of scale obtained in this study may not be as accurate as required, on specific situation. The futures studies can also contribute to Persian older people with other climatic conditions, various health statuses and living circumstances. Moreover, the convergent and discriminant validity were not investigated among older adult in this study.

## Conclusions

Based on the results PASE had an acceptable translation, validity, and reliability in the Persian language. Due to the increasing population of the elderly, countries are now trying to improve the health of the elderly and pay attention to their well-being, it is necessary to assess and evaluate physical activity. PASE is a simple and easy scale that can use to evaluate the physical activity of the elderly in a few minutes and self-report.

## Data Availability

The datasets used and/or analyzed during the current study are available from the corresponding author on reasonable request.

## Abbreviations

ADL: Activities of Daily Living
AGFI: Adjusted Goodness of Fit Index
AMOS: Analysis of Moment Structure
CFA: Confirmatory Factor Analysis
CFI: Comparative Fit Index
CMIN/DF: Minimum Discrepancy Function by Degrees of Freedom Divided
COSMIN: Consensus-Based Standards for the Selection of Health Status Measurement Instruments
CVI: Content Validity Index
CVR: Content Validity Rate
DF: Degree of Freedom
FMI: Functional Independence Measure
ICC: Intraclass Correlation Coefficient
IFI: Incremental Fit Index
IS: Impact Score
GFI: Goodness of Fit Index
NFI: Normed Fit Index
NNFI: Non-Normed Fit Index
P: *P*-Value
PASE: Physical Activity Scale for the Elderly
PNFI: Parsimonious Normed Fit Index
RMR: Root Mean Square Residual
RMSEA: Root Mean Square Error of Approximation
SPSS: Statistical Package for the Social Sciences
χ2: Chi-Square
χ2/df: Ratio of Chi Square to Degrees Of Freedom

## References

[1] Taasim S. Ageing population and economic growth: evidence from Malaysia. South Asian J Soc Stud Eco. 2020;7(4):11–8.

[2] Langhammer B, Bergland A, Rydwik E. The importance of physical activity exercise among older people. BioMed Res Int. 2018;2018:1–3. 10.1155/2018/7856823 PMC6304477 30627571

[3] Karimi Z, Majlesi F, Tol A, Rahimi Foroushani A, Sahaf R, Ali Gol M, Mohebbi S (2015). The Effect of Educational Intervention on the Promotion of Physical Activities of the Elderly Men in Qom City: Application of Trans-Theoretical Model. Salmand Iran J Ageing.

[4] Achttien RJ, van Lieshout J, Wensing M, Nijhuis-van der Sanden M, Staal JB (2020). The decline in physical activity in aging people is not modified by gender or the presence of cardiovascular disease. Eur J Pub Health.

[5] Hosseini FS, Hossein zadeh R (2011). Effect of physical activity on physical and mental health in elderly men. J Health Care.

[6] Rezaei s, Esmaeili M (2017). The effect of physical activities on the quality of life, hope and life satisfaction among the elderly in Ilam city. J Gerontol.

[7] Colpani V, Oppermann K, Spritzer PM (2013). Association between habitual physical activity and lower cardiovascular risk in premenopausal, perimenopausal, and postmenopausal women: a population-based study. Menopause.

[8] Kalani Z, Pourmovahed Z, Farajkhoda T, Bagheri I (2018). A Qualitative Approach to Women’s Perspectives on Exercise in Iran. Int J Commun Based Nurs Midwifery.

[9] Denkinger MD, Franke S, Rapp K, Weinmayr G, Duran-Tauleria E, Nikolaus T, Peter R (2010). Accelerometer-based physical activity in a large observational cohort-study protocol and design of the activity and function of the elderly in Ulm (ActiFE Ulm) study. BMC Geriatr.

[10] Salehi L, Shokrvash B, Jamshidi E, Montazeri A (2014). Physical activity in Iranian older adults who experienced fall during the past 12 months. BMC Geriatr.

[11] Saint Martin M, Sforza E, Barthélémy J, Roche F, Lefèvre P, Liénard G, Thomas-Anterion C (2017). Long-lasting active lifestyle and successful cognitive aging in a healthy elderly population: The PROOF cohort. Rev Neurol.

[12] Jansen C-P, Claßen K, Hauer K, Diegelmann M, Wahl H-W (2014). Assessing the effect of a physical activity intervention in a nursing home ecology: a natural lab approach. BMC Geriatrics.

[13] Lok N, Lok S, Canbaz M (2017). The effect of physical activity on depressive symptoms and quality of life among elderly nursing home residents: Randomized controlled trial. Arch Gerontol Geriatr.

[14] Lerche S, Gutfreund A, Brockmann K, Hobert MA, Wurster I, Sünkel U, Eschweiler GW, Metzger FG, Maetzler W, Berg D (2018). Effect of physical activity on cognitive flexibility, depression and RBD in healthy elderly. Clin Neurol Neurosurg.

[15] Lee HJ, Kim KD (2018). Effect of physical activity on cognition and daily living activities of the elderly with mild dementia. J Phys Ther Sci.

[16] Goethals L, Barth N, Guyot J, Hupin D, Celarier T, Bongue B (2020). Impact of home quarantine on physical activity among older adults living at home during the COVID-19 pandemic: qualitative interview study. JMIR Aging.

[17] McGlinchey MP, James J, McKevitt C, Douiri A, McLachlan S, Sackley CM (2018). The effect of rehabilitation interventions on physical function and immobility-related complications in severe stroke—protocol for a systematic review. Syst Rev.

[18] Plouvier S, Gourmelen J, Chastang J-F, Lanoë J-L, Leclerc A (2011). Low back pain around retirement age and physical occupational exposure during working life. BMC Public Health.

[19] Laurent M, Oubaya N, David J-P, Engels C, Canoui-Poitrine F, Corsin L, Liuu E, Audureau E, Bastuji-Garin S, Paillaud E (2020). Functional decline in geriatric rehabilitation ward; is it ascribable to hospital acquired infection? A prospective cohort study. BMC Geriatr.

[20] Arik G, Varan HD, Yavuz BB, Karabulut E, Kara O, Kilic MK, Kizilarslanoglu MC, Sumer F, Kuyumcu ME, Yesil Y (2015). Validation of Katz index of independence in activities of daily living in Turkish older adults. Arch Gerontol Geriatr.

[21] Martin KA, Rejeski WJ, Miller ME, James MK, Ettinger WHJ, Messier SP (1999). Validation of the PASE in older adults with knee pain and physical disability. Med Sci Sports Exerc.

[22] Sattler MC, Jaunig J, Tösch C, Watson ED, Mokkink LB, Dietz P, van Poppel MNM (2020). Current Evidence of Measurement Properties of Physical Activity Questionnaires for Older Adults: An Updated Systematic Review. Sports Med.

[23] Sun F, Norman IJ, While AE (2013). Physical activity in older people: a systematic review. BMC Public Health.

[24] Moschny A, Platen P, Klaaßen-Mielke R, Trampisch U, Hinrichs T (2011). Physical activity patterns in older men and women in Germany: a cross-sectional study. BMC Public Health.

[25] Washburn RA, Smith KW, Jette AM, Janney CA (1993). The Physical Activity Scale for the Elderly (PASE): development and evaluation. J Clin Epidemiol.

[26] Ayvat E, Kilinc M, Kirdi N (2017). The Turkish version of the Physical Activity Scale for the Elderly (PASE): its cultural adaptation, validation, and reliability. Turkish J Med Sci.

[27] Ngai SP, Cheung RT, Lam PL, Chiu JK, Fung EY (2012). Validation and reliability of the Physical Activity Scale for the Elderly in Chinese population. J Rehabil Med.

[28] Organization WH. Process of translation and adaptation of instruments. https://wwww.hoint/substance_abuse/research_tools/translation/en/ 2021.

[29] Covotta A, Gagliardi M, Berardi A, Maggi G, Pierelli F, Mollica R, Sansoni J, Galeoto G. Physical activity scale for the elderly: translation, cultural adaptation, and validation of the Italian version. Curr Gerontol Geriatr Res. 2018;2018:1–7. 10.1155/2018/8294568 PMC6129314 30224917

[30] Mohajan HK (2017). Two criteria for good measurements in research: Validity and reliability. Ann Spiru Haret Univ Econ Ser.

[31] Keszei AP, Novak M, Streiner DL (2010). Introduction to health measurement scales. J Psychosom Res.

[32] McDowell I. Measuring health: a guide to rating scales and questionnaires, Third ed. New York: Oxford University Press, USA; 2006.

[33] Ghanbari-Homayi S, Dencker A, Fardiazar Z, Jafarabadi MA, Mohammad-Alizadeh-Charandabi S, Meedya S, Mohammadi E, Mirghafourvand M: Validation of the Iranian version of the childbirth experience questionnaire 2.0. BMC Pregnancy Childbirth 2019;19(1):465. 10.1186/s12884-019-2606-y PMC6894263 31801477

[34] Halvani A, Pourfarokh N, Nasiriani K. Quality of life and related factors in patients with chronic obstructive pulmonary disease. 2006.

[35] Polit DF, Beck CT, Polit D: Resource manual for nursing research. Generating and Assessing evidence for nursing practice 9th ed China: Wolters Kluwer Health 2012.

[36] Juniper EF, Guyatt GH, Streiner DL, King DR (1997). Clinical impact versus factor analysis for quality of life questionnaire construction. J Clin Epidemiol.

[37] Yusoff MSB (2019). ABC of content validation and content validity index calculation. Resource.

[38] Zamanzadeh V, Ghahramanian A, Rassouli M, Abbaszadeh A, Alavi-Majd H, Nikanfar A-R (2015). Design and Implementation Content Validity Study: Development of an instrument for measuring Patient-Centered Communication. J Caring Sci.

[39] Ayre C, Scally AJ (2014). Critical values for Lawshe’s content validity ratio: revisiting the original methods of calculation. Measurement Evaluation in Counseling Development.

[40] Rodrigues IB, Adachi JD, Beattie KA, MacDermid JC (2017). Development and validation of a new tool to measure the facilitators, barriers and preferences to exercise in people with osteoporosis. BMC Musculoskelet Disord.

[41] Baradaran Eftekhari M, Dejman M, Forouzan AS, Falahat K, Shati M, Mirabzadeh A, Bass J, Mahmoodi Z. Developing a Depression Inventory for Screening the Fars Ethnicity in Iran. Iran J Psychiatry Behav Sci. 2019;13(3):1–7.

[42] Ibiyemi A, Mohd Adnan Y, Daud MN, Olanrele S, Jogunola A (2019). A content validity study of the test of valuers’ support for capturing sustainability in the valuation process in Nigeria. Pacific Rim Property Res J.

[43] Brown TA, Moore MT. Confirmatory factor analysis. Handbook of structural equation modeling 2012:361–379.

[44] Ismail N, Hairi F, Choo WY, Hairi NN, Peramalah D, Bulgiba A (2015). The Physical Activity Scale for the Elderly (PASE):Validity and Reliability Among Community-Dwelling Older Adults in Malaysia. Asia Pacific J Public Health.

[45] Granger CL, Parry SM, Denehy L (2015). The self-reported Physical Activity Scale for the Elderly (PASE) is a valid and clinically applicable measure in lung cancer. Supp Care Cancer.

[46] Hagiwara A, Ito N, Sawai K, Kazuma K (2008). Validity and reliability of the Physical Activity Scale for the Elderly (PASE) in Japanese elderly people. Geriatr Gerontol Int.

[47] Svege I, Kolle E, Risberg MA (2012). Reliability and validity of the Physical Activity Scale for the Elderly (PASE) in patients with hip osteoarthritis. BMC Musculoskelet Disord.

